# A Seven Toes Foot: Case Series as an Isolated Dysplasia With Variety of Appearance

**DOI:** 10.7759/cureus.32949

**Published:** 2022-12-26

**Authors:** Nickolaos Laliotis, Panagiotis Konstantinidis, Chrysanthos Chrysanthou

**Affiliations:** 1 Orthopaedics, Interbalkan Medical Center, Thessaloniki, GRC; 2 Anatomy and Surgical Anatomy, Aristotle University of Thessaloniki, Thessaloniki, GRC

**Keywords:** foot polydactyly, mirror foot, foot congenital dysplasia, 7 toe foot, complex limb dysplasia

## Abstract

A seven toes foot is an extremely rare foot disorder. It may appear as part of a complex limb dysplasia or as an isolated deformity. We present five children with a seven toes foot. Supernumerary bones of the foot were found either as isolated duplicated great and little toe either affecting proximal bones, including the metatarsals or midtarsal bones, characterized as mirror foot.

Radiological examination with X-rays was adequate for the evaluation of the affected foot. All patients had dysplasia isolated to the foot. Spine dysplasia was found in one child, and a hypoplastic unilateral little finger in another. The affected foot had a plantigrade shape without leg length discrepancy (LLD). The aesthetic of the enlarged foot with the problems of shoe wear was the main concern of the dysplasia.

Surgical treatment was provided after the walking age. Surgery was demanded to provide a cosmetically acceptable foot. Removal of the supernumerary rays, either the medial or the central rays, was performed after the radiological evaluation. Our results were satisfactory, and none of our patients required additional interventions, although a mild varus position of the great toe was still observed in one patient.

## Introduction

Polydactyly of the foot is a common disorder. But the expression of dysplasia with a seven ray foot is rare. Patterns of polydactyly are described as preaxial, postaxial, or central. Polydactyly may affect only the toes or may be consisted of an increased number of proximal bones of the foot, metatarsals, cuneiforms, or tarsal bones. Mirror foot is an extremely rare congenital anomaly characterized by a preaxial increased number of foot rays that have anatomical characteristics of postaxial rays. Occasionally it is associated with proximal skeletal anomalies of the fibula or tibia, either deficiency or duplication of the remaining bone. The mirror foot consists of seven or more toes; it is also described as preaxial or central. It may be part of a syndrome such as VACTERL, Martin, Lauren Sandrow, or fibular dimelia [1-9].

We present five children with seven ray feet. In all but one of them, the dysplasia was unilateral. The dysplasia was affecting only the foot and was not correlated with a syndrome. In all patients, the affected foot had a plantigrade shape. No leg length discrepancy (LLD) was found. One child had associated vertebra, rib congenital anomalies, and another had a unilateral hypoplastic little finger. There was a wide variety of types of seven toes polydactyly. It was affecting either only the toes with pre- and post-axial lesions or affecting metatarsal and tarsal bones with preaxial or central mirror type of foot. Surgical treatment was demanding, with the aim to reduce the width of the foot for appropriate shoe wear. It is important to retain muscles and tendons that are attached to supernumerary bones, to maintain the foot function. The rare foot dysplasia appears in our children as an isolated dysplasia, with normal skeletal development of the limb. In cases of prenatal diagnosis of foot polydactyly, as an isolated dysplasia, parents can be informed about the appropriate treatment of the dysplasia.

## Case presentation

During the last 25 years, we have treated five children with a seven-ray foot. Among a series of more than 40 children with six-ray feet, we recruited these children from our clinical data. This is a retrospective study, having the approval of the Interbalkan Medical Center ethical committee on 26/04/2022. Our study is based on photographic evidence, X-rays, follow-up pictures, and results from clinical notes of the children. All children were of Caucasian origin. Dysplasia was apparent at birth, while in three of our children, diagnosis of polydactyly of the foot was detected prenatally.

Children were first evaluated in our referral center for pediatric orthopedics at ages that ranged between five months to one year old. The child with bilateral seven toes was referred at two years old. A detailed examination consisted of the assessment of the presence of any other bone dysplasia. Upper limbs, spine, and lower limbs were evaluated for discrepancies in length and transverse diameter. Plain X-rays were used to examine the supernumerary bones of the affected foot. Ultrasound examination of the genitourinary system was performed in all children, and all were referred to with normal measurements.

All children had normal motor development and started walking at the age of one year with a range of three months. The aesthetic picture of the foot was the main concern of the parents, with the difficulties to accommodate a normal shoe. All four children, apart from the child with bilateral polydactyly, had to use different sizes of shoes. None of the affected limbs had leg length discrepancy.

The third child had multiple congenital hemivertebra with congenital scoliosis and asymmetry of the ribs that were treated simultaneously with a brace. We achieved an impressive correction of the shape of his body, avoiding the progression of scoliosis. None of our patients had bone dysplasia of the affected limb, apart from the foot polydactyly.

The radiological examination consisted of plain X-rays of the feet and tibia. We examined the level of increased rays and the location of the multiple rays, describing pre-, central, or post-axial multiple rays (Table 1).

**Table 1:** Description of the patients.

Patient	Sex	Age at the initial examination	Other dysplasias	X-ray findings	Type of polydactyly	Follow up
1st	Male	5 months	Vertebral and costal dysplasia	7 toes, 6 metatarsals, extra cuneiform, extra navicular. Left foot	Preaxial	9 years
2nd	Male	1 2 months	No other dysplasia	7 toes, 7 metatarsals. Left foot	Central	3 years
3rd	Male	6 months	No other dysplasia	7 toes, 7 metatarsals. Left foot	Preaxial	8 years
4th	Male	24 months	Supernumerary 5^th^ finger hypoplastic	Bilateral. 5 metatarsals 7 toes (great and little toe).	Unspecified	2 years
5th	Male	6 months	No other dysplasia	7 toes, 6 metatarsals, possible extra cuneiform. Right foot	Preaxial	1/2 year

**Figure 1:**
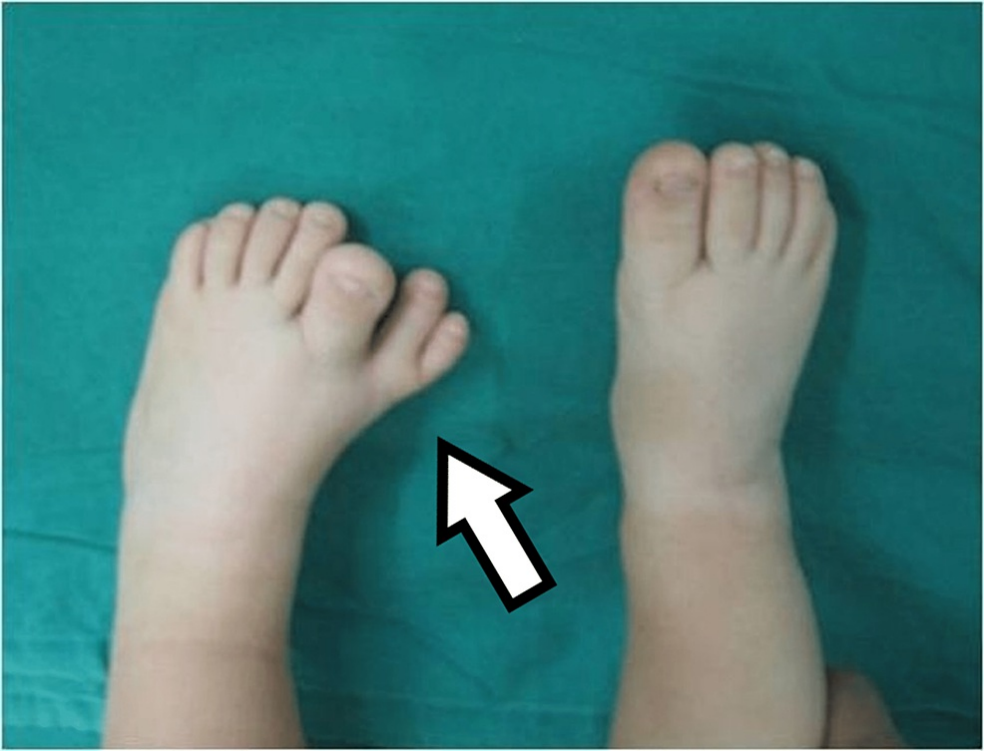
Dorsal view of the first patient's affected foot.

**Figure 2:**
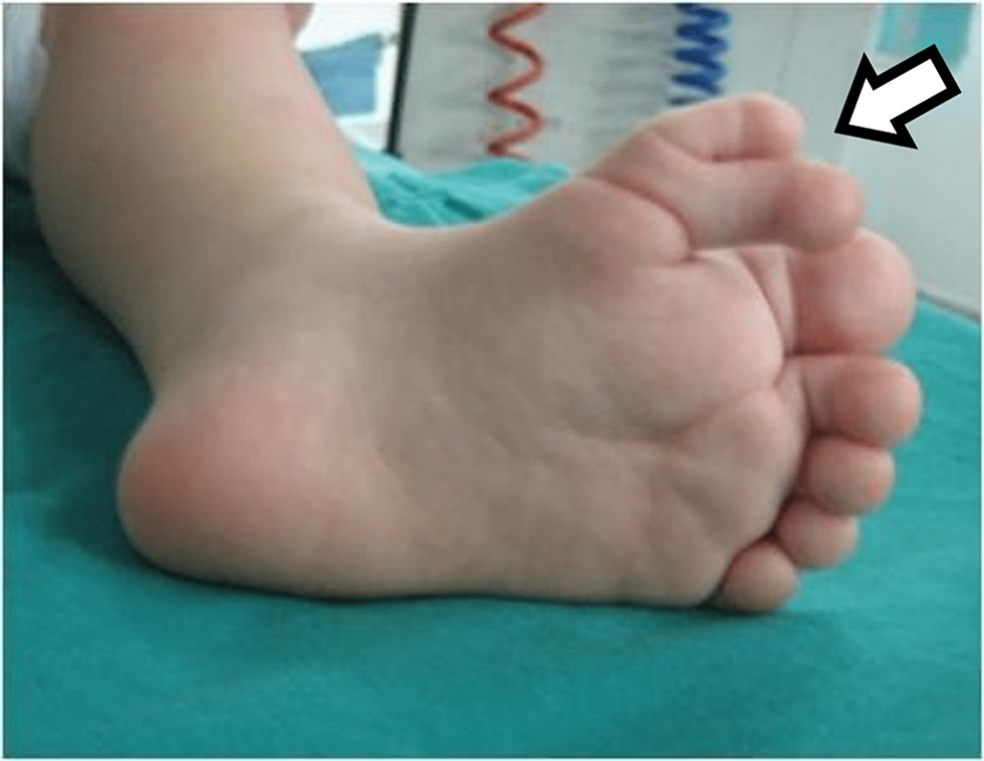
Plantar view of the first patient's affected foot.

**Figure 3:**
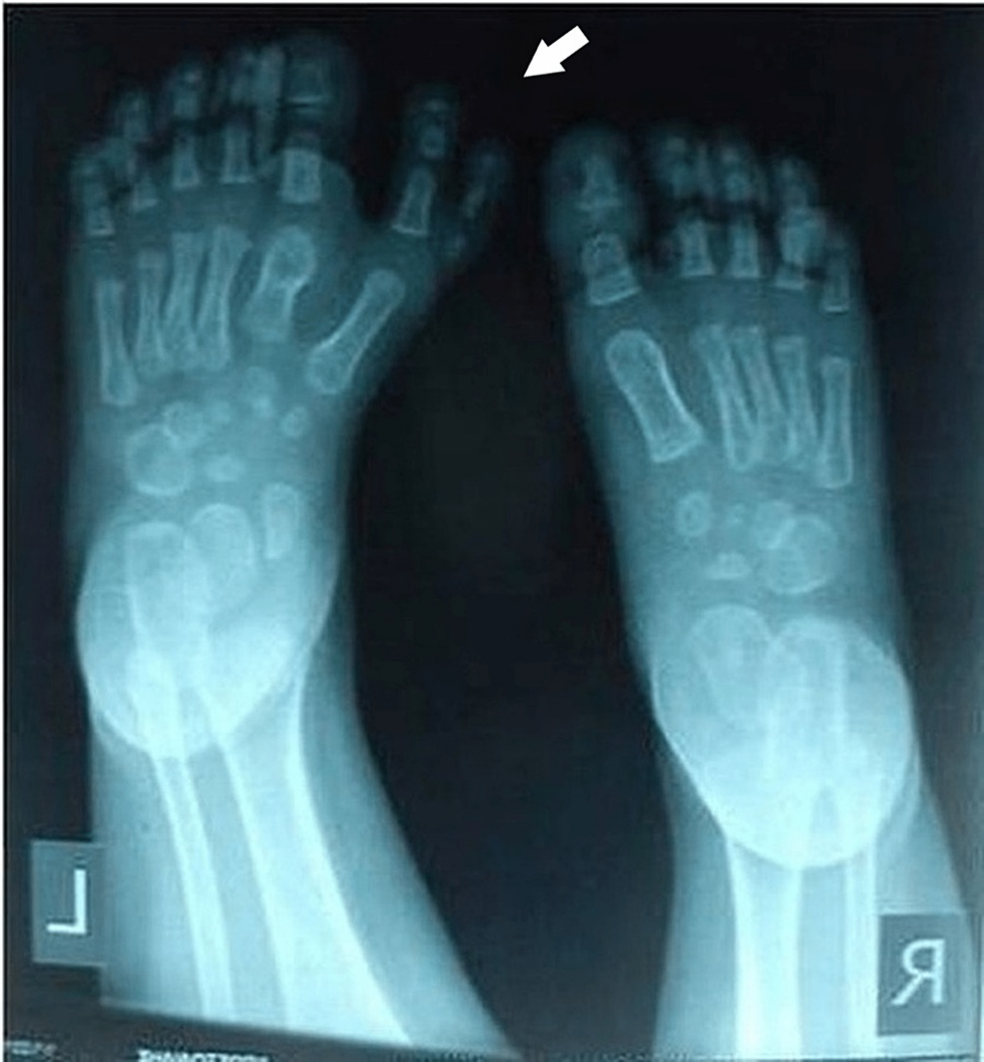
X-ray of the first patient's affected foot. X-ray with duplication of the medial rays, with both supernumerary toes having three phalanges, with hypoplastic the medial part. There were six metatarsals, supernumerary cuneiforms, and hypoplastic supernumerary navicular. The second metatarsal, corresponding to the normal great toe, had a proximal position of the epiphysis.

The first patient had preaxial polydactyly, with seven toes, with duplication of the medial rays, with both supernumerary toes having three phalanges with hypoplastic the medial part, while the third toe was the great toe and appeared with two phalanges. There were six metatarsals, supernumerary cuneiforms, and hypoplastic supernumerary navicular. The second metatarsal, corresponding to the normal great toe, had a proximal position of the epiphysis (Figures 1-3).

**Figure 4:**
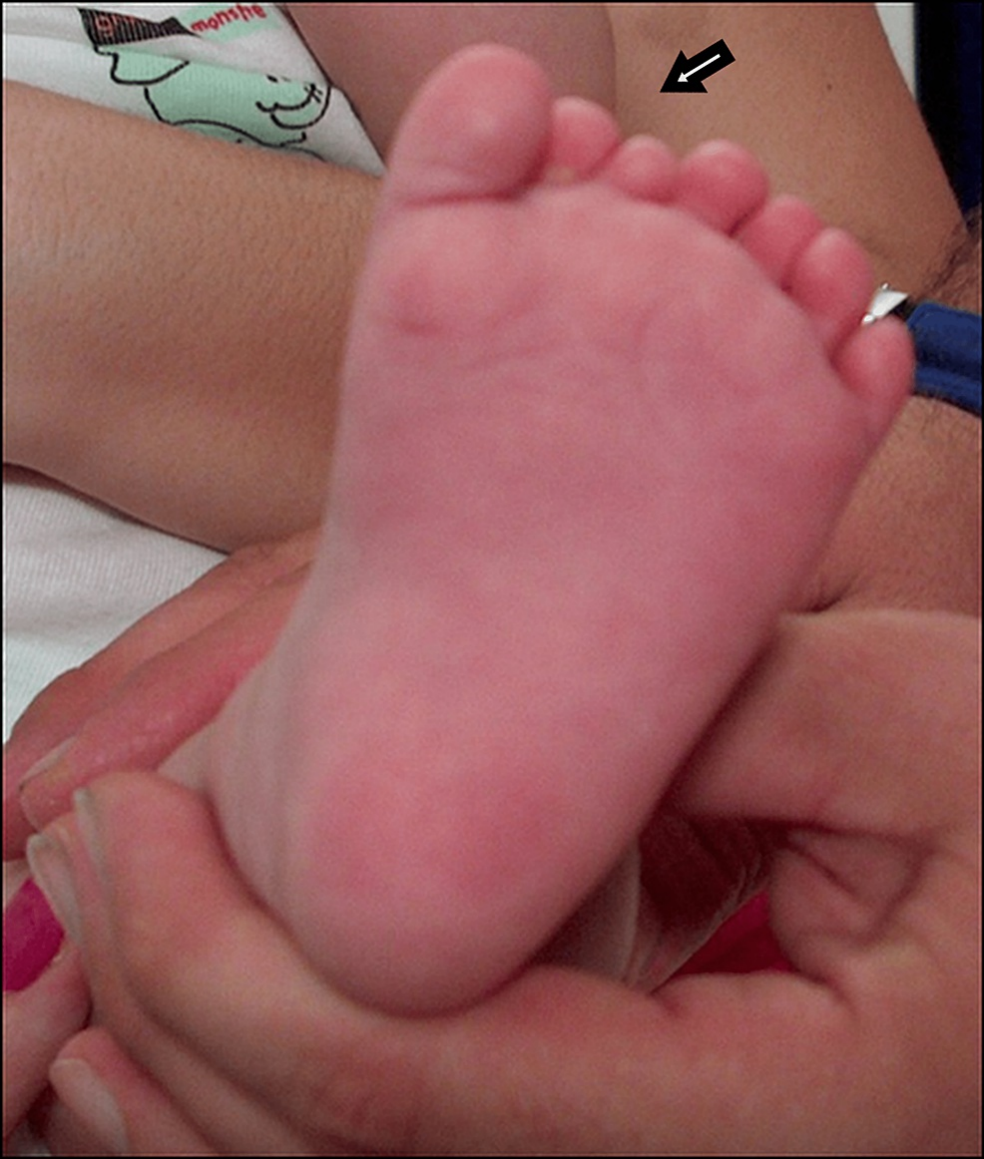
Plantar view of the second patient's mirror foot with central polydactyly.

**Figure 5:**
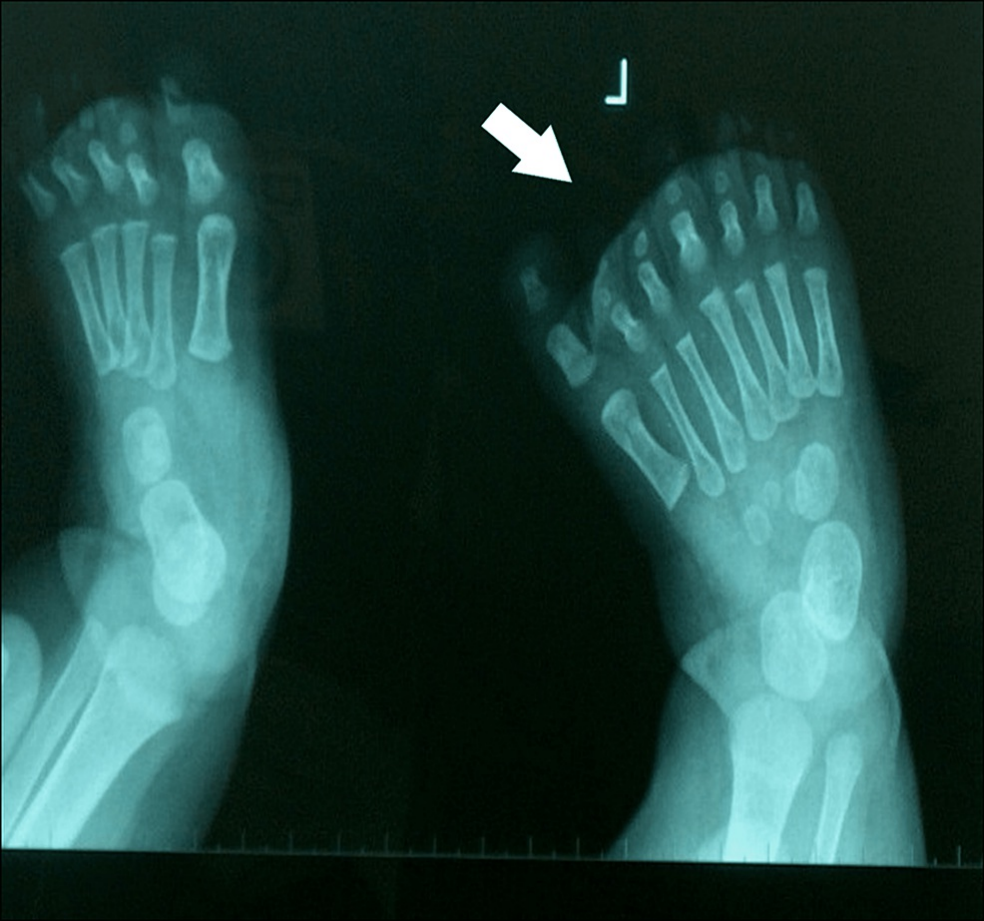
X-ray of the second patient's mirror foot with central polydactyly.

The second child had a mirror foot with a central polydactyly, with normal development of the medial ray, with the shape of the metatarsal as expected for the medial ray, and with a toe with two phalanges. It was not articulating with the cuneiforms. There were three cuneiforms. Metatarsals and toes in the middle (second and third) had normal development. In total, there were seven toes and metatarsals (Figures 4, 5).

**Figure 6:**
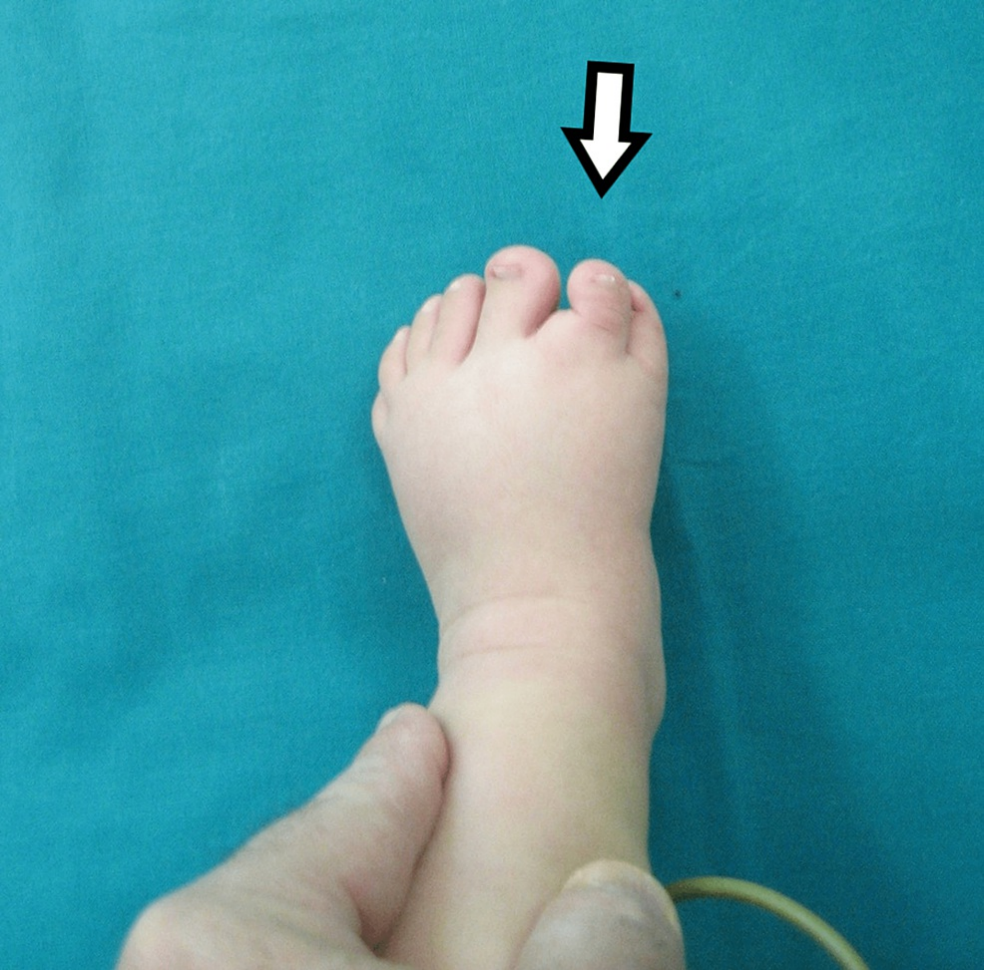
Dorsal view of the third child, with medial mirror foot.

**Figure 7:**
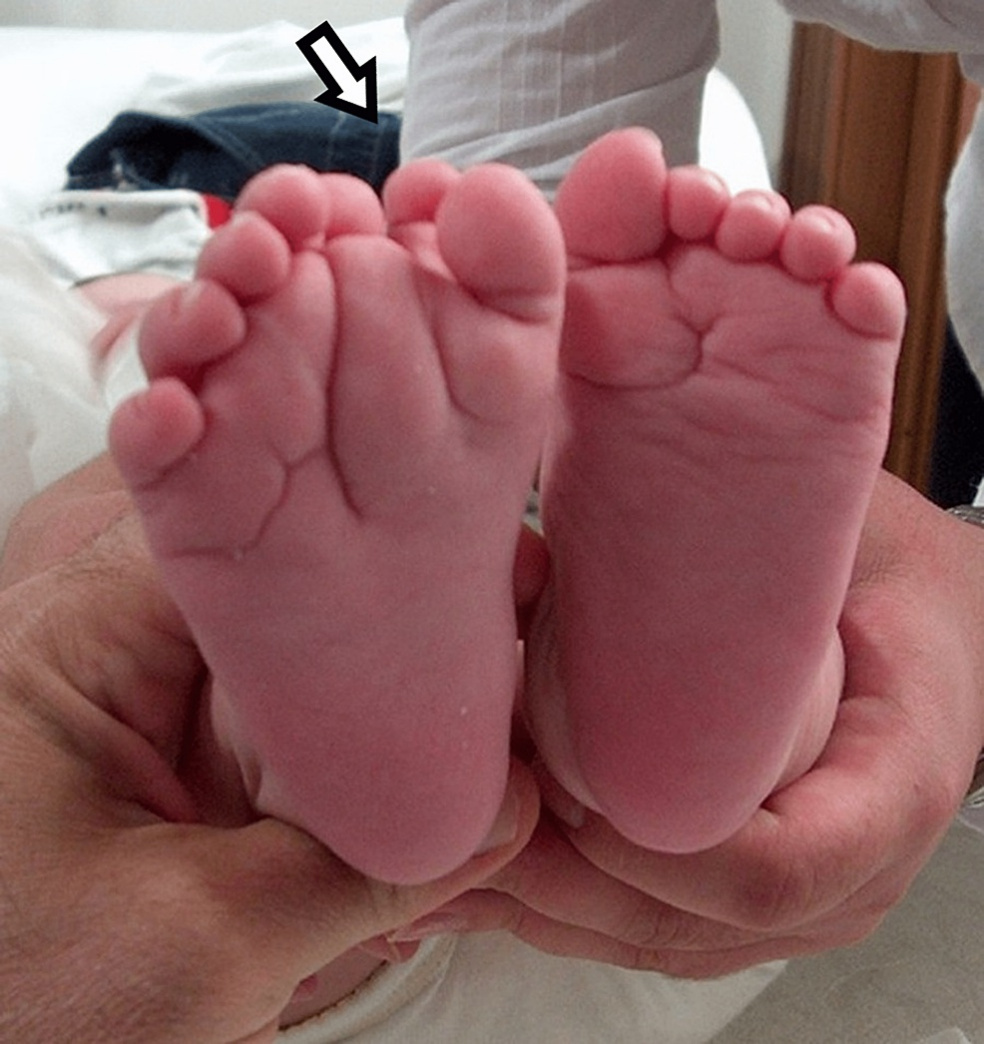
Plantar view of the third child, with medial mirror foot.

**Figure 8:**
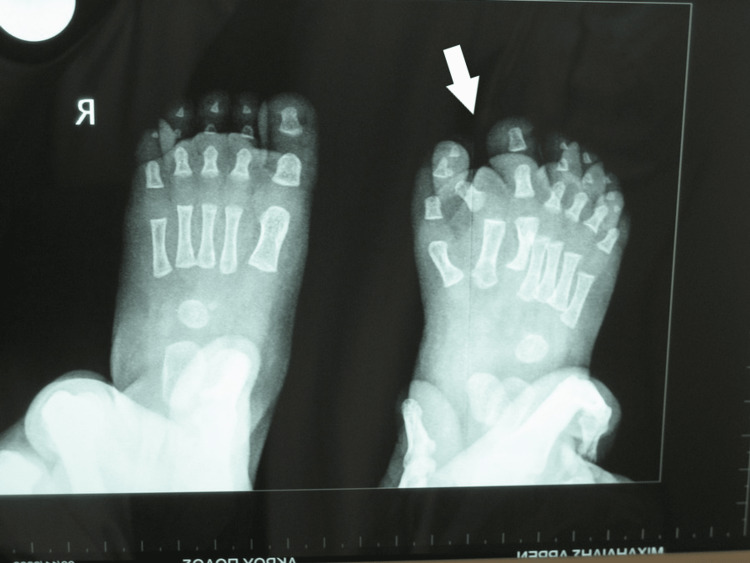
X-ray of the third patient's affected foot. X-ray of the foot with the 3d ray with a toe with two phalanges. The medial supernumerary toes, first, and second appeared enlarged and with a deviation of the axis. The remaining 4-7 metatarsals and toes on the lateral side appeared with normal shape. Cuneiforms and tarsal bones appeared normal on the initial X-ray.

The third child had a medial ray polydactyly with seven metatarsals. The 3d ray had a toe with two phalanges. The medial supernumerary toes, first and second appeared enlarged and with a deviation of the axis. The remaining 4-7 metatarsals and toes on the lateral side appeared with normal shape. Cuneiforms and tarsal bones appeared normal on the initial X-ray (Figures 6-8).

**Figure 9:**
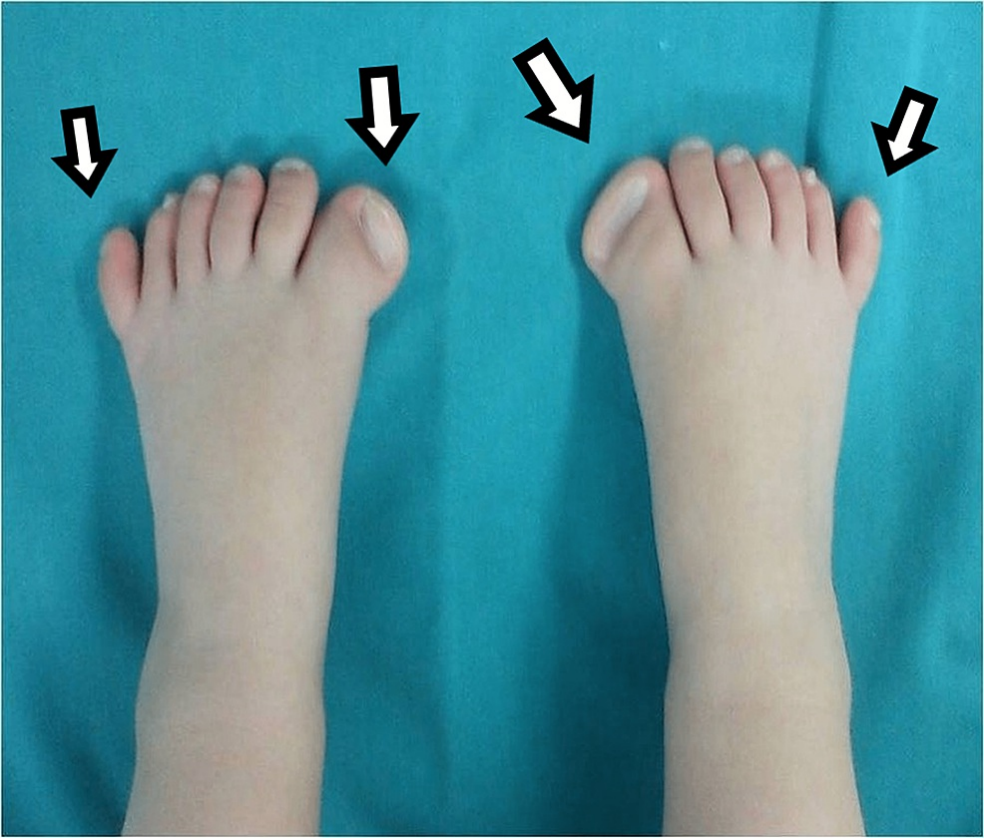
Picture of the fourth patient, with bilateral involvement affecting both the great toe and the little toe.

**Figure 10:**
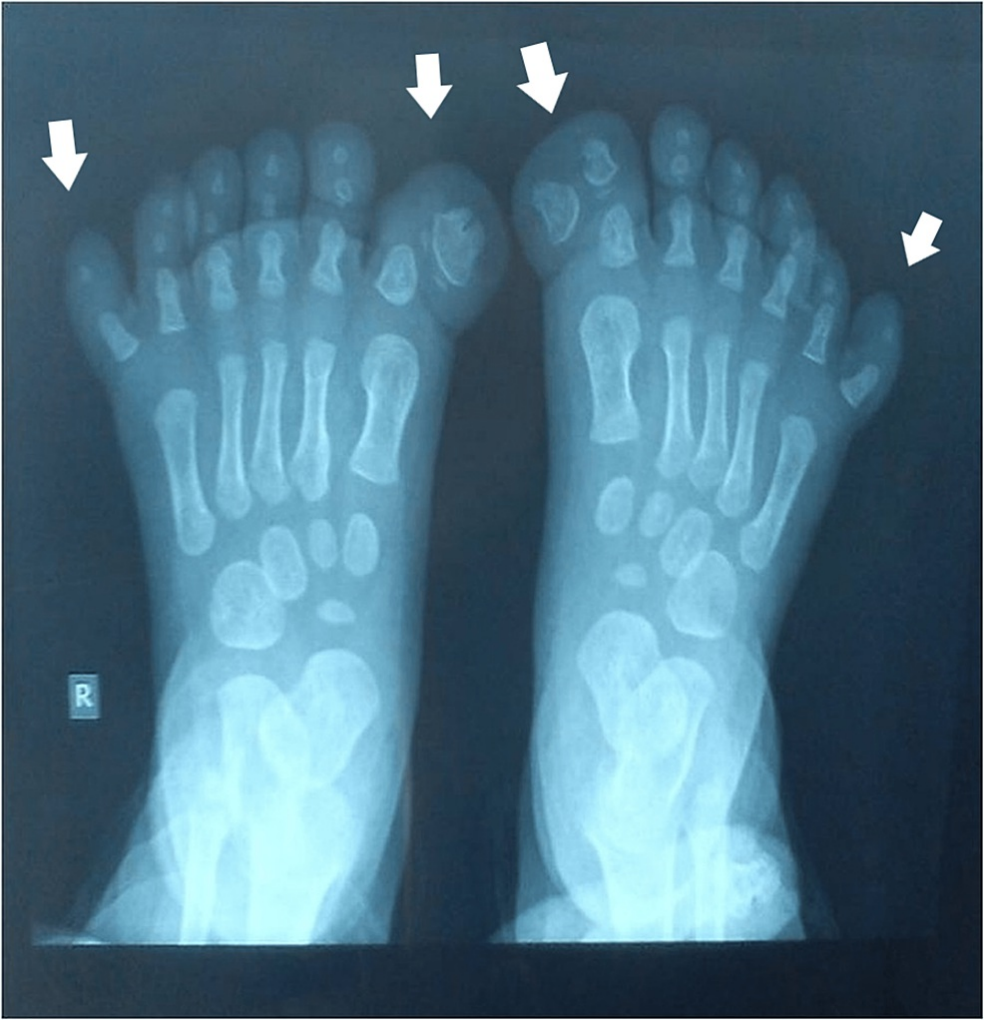
X-ray of the fourth patient's affected feet. X-ray of the feet with seven toes affecting both the medial and the lateral ray, with normal five metatarsals. Big toes that were duplicated were fused on the right side and had a varus position, while the nail was a broad one. On the left side, there was a single racket-type phalange and a developed two phalanges toe.

The fourth child had bilateral involvement. He had a supernumerary hypoplastic fifth finger, united with a thin skin fold in the ulnar side of the left hand. Feet appeared with seven toes affecting both the medial and the lateral ray, with normal five metatarsals. Big toes that were duplicated were fused on the right side and had a varus position, while the nail was a broad one. On the left side, there was a single racket-type phalange and a developed two phalanges toe. The lateral toe was connected with normal skin to the side of the foot (Figures 9, 10).

**Figure 11:**
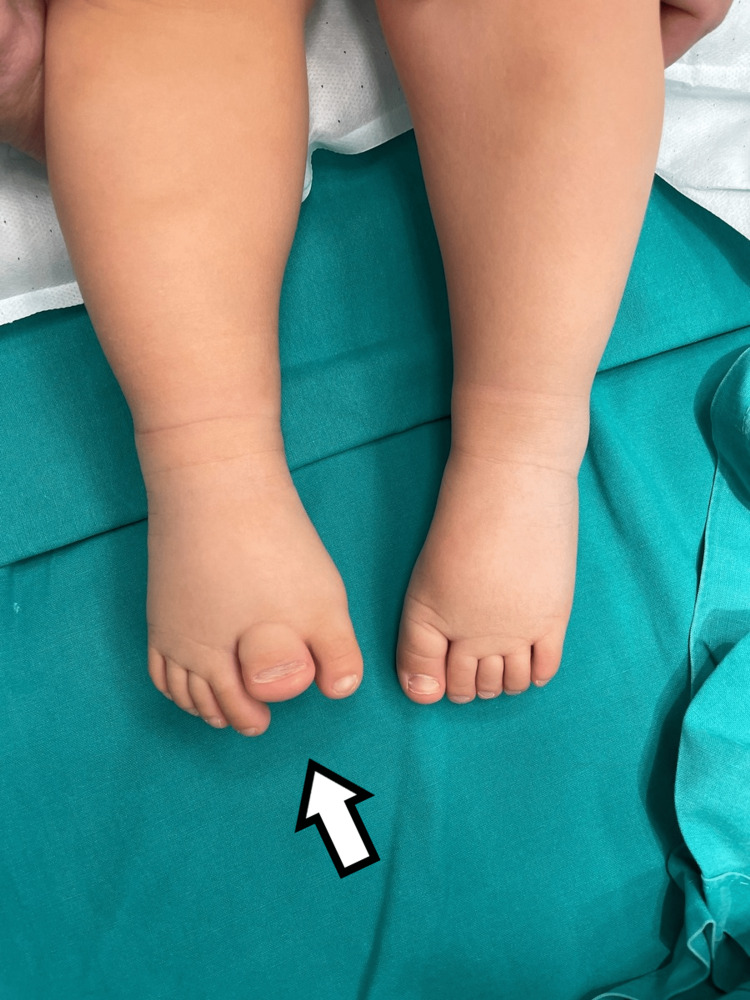
Dorsal view of the fifth patient.

**Figure 12:**
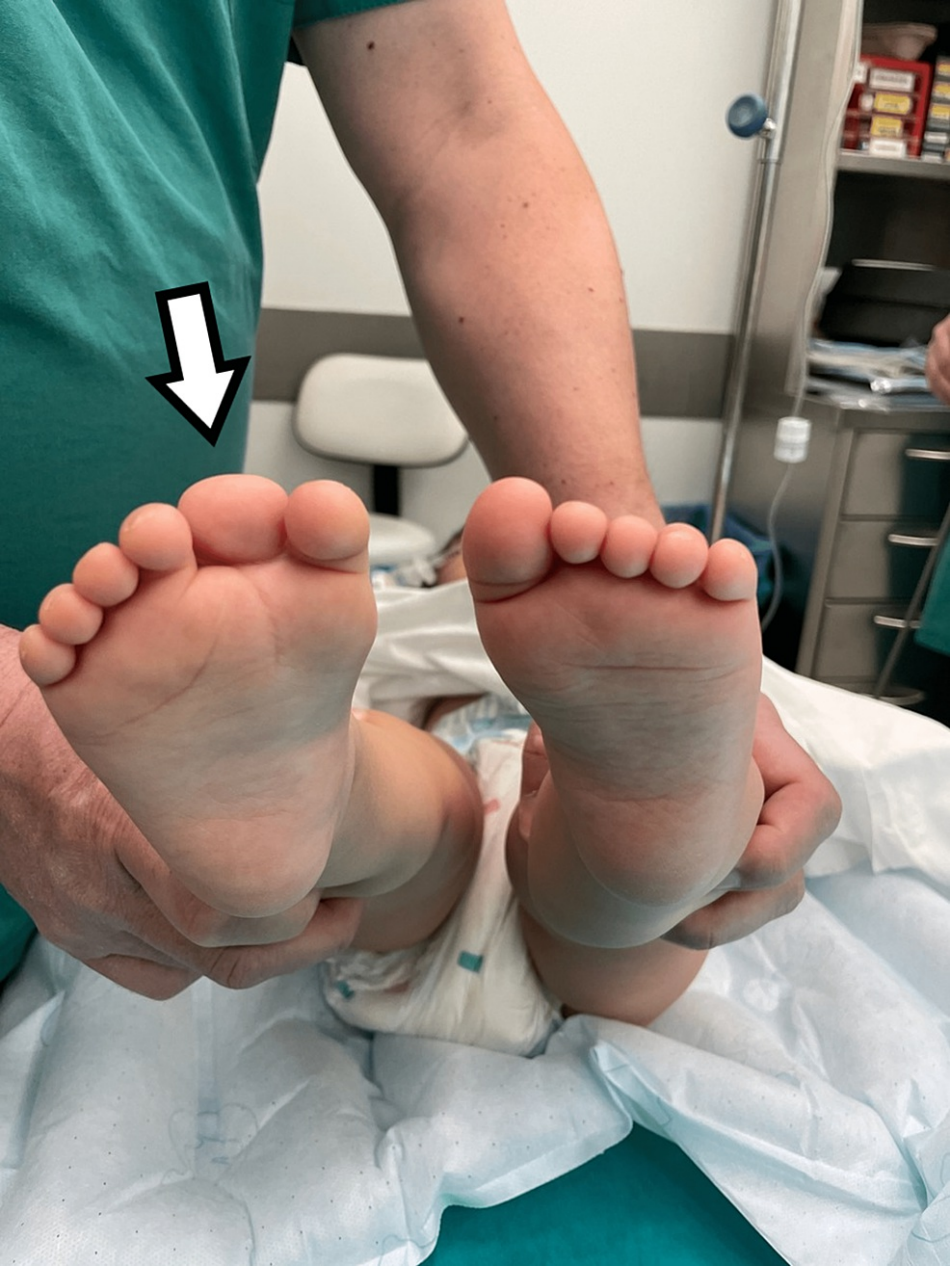
Plantar view of the fifth patient.

**Figure 13:**
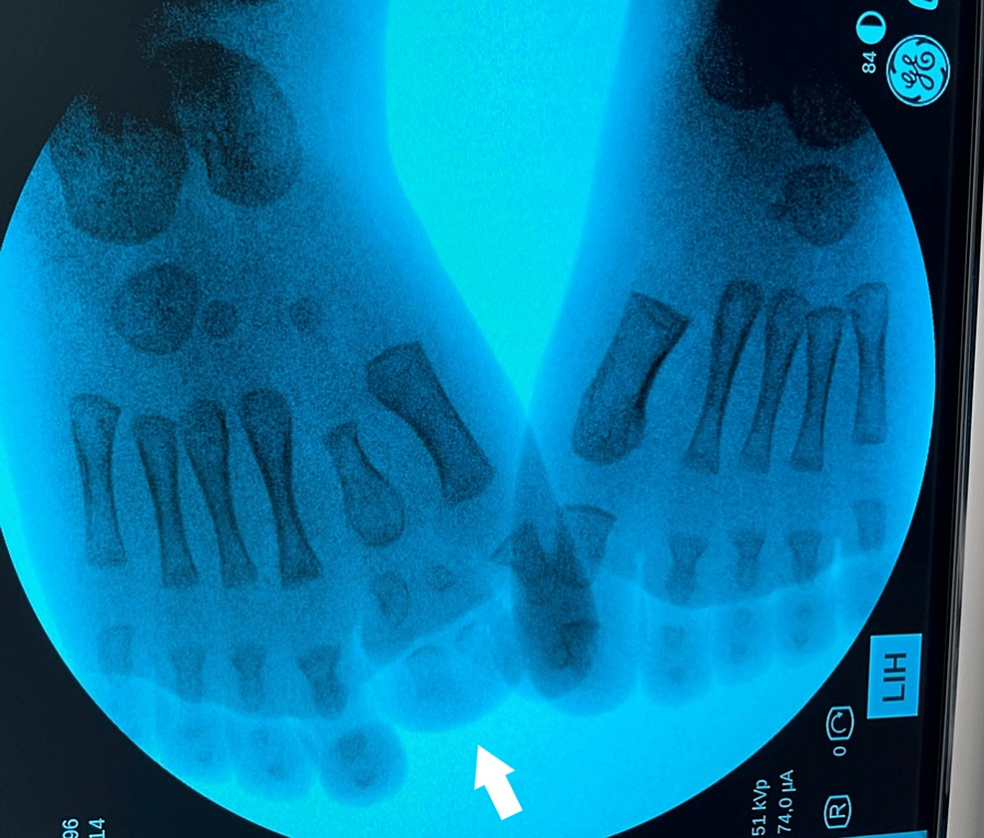
X-ray of the fifth patient. X-ray with a medial duplication with six metatarsals. The second hypoplastic metatarsal is articulating with two toes. They were fused with a central crease of the toenail.

**Figure 14:**
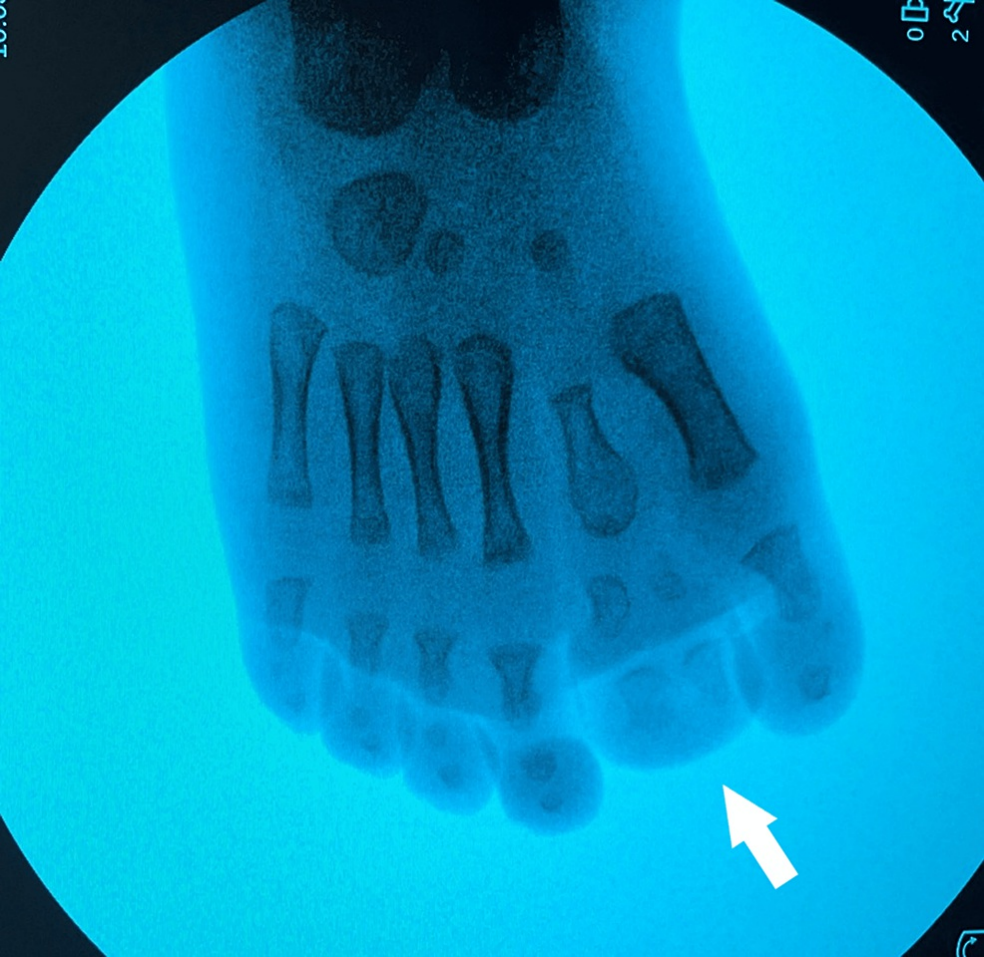
X-ray of the fifth patient's affected foot. X-ray with a medial duplication with six metatarsals. The second hypoplastic metatarsal is articulating with two toes. They were fused with a central crease of the toenail.

The fifth child had a medial duplication with six metatarsals, with the second one similar to the metatarsal of the great toe, with two toes corresponding to the supernumerary metatarsal that was fused with a central crease of the toenail (Figures 11-14).

All children appeared with a plantigrade foot, with a normal shape and normal movements of the ankle and subtalar joint.

After the appropriate consultation with the parents, surgical treatment was provided to all our patients in order to reduce the size of the foot and restore the best possible aesthetic shape of the foot. Surgery is performed after the walking age.

Results: Surgical treatment for the four children, with the increased number of metatarsals, either six or seven, was the removal of the supernumerary rays, keeping in place the ray that was scheduled as the medial ray of the great toe.

**Figure 15:**
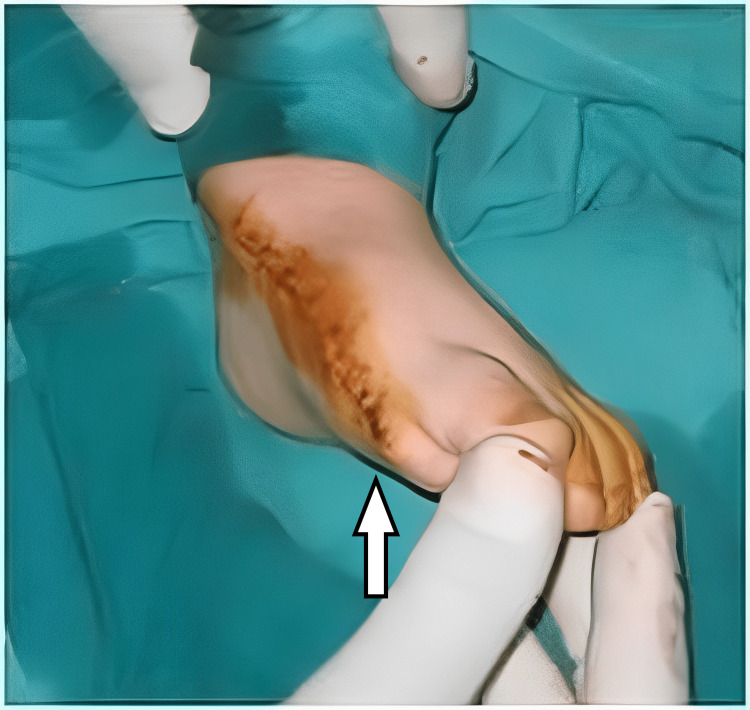
The appearance of the first patient's foot immediately after the procedure.

For the first patient, the medial metatarsal was removed with the distal duplication of the toes up to the cuneiform and supernumerary navicular. On surgical exploration, the tibialis posterior was found completely attached to the extra navicular, and the decision was not to sacrifice the navicular to keep the normal strength of the foot. The metatarsal that was left had articulated the toe with two phalanges (Figure 15).

**Figure 16:**
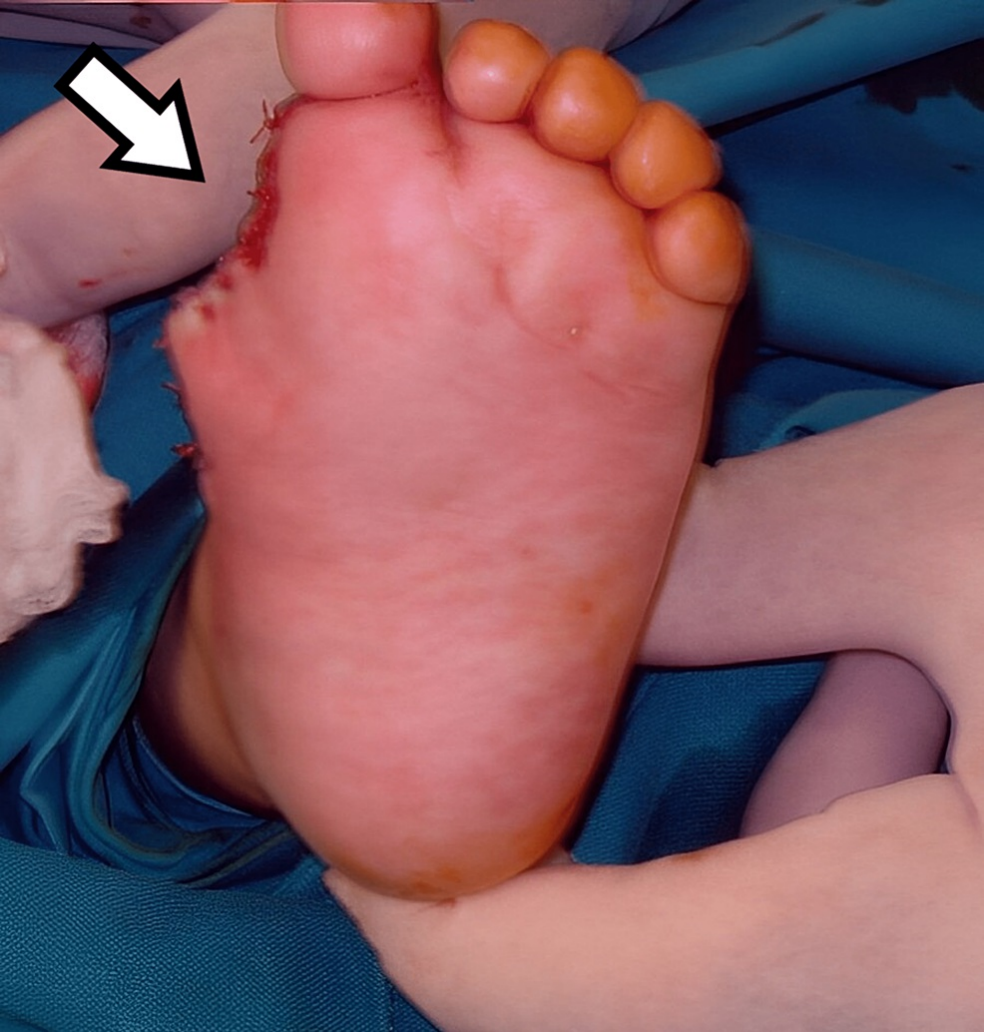
The picture of the third patient's foot after the procedure.

The same approach with surgical removal of the medial supernumerary rays was performed for the third patient (Figure 16).

**Figure 17:**
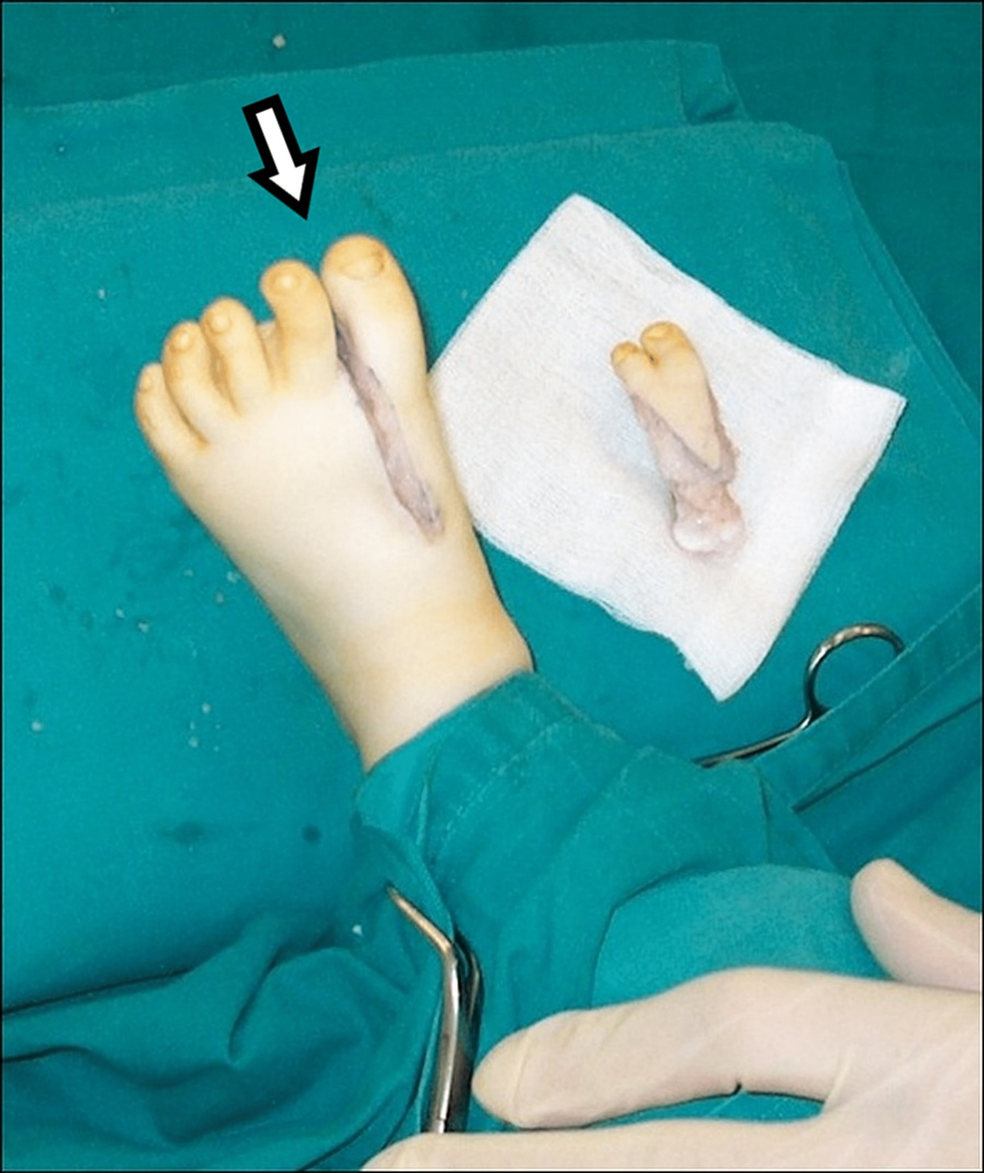
Intraoperative picture of the second patient. Intraoperative picture of the two central metatarsals and toes that were removed and the immediate intraoperative picture of the foot of the second patient.

For the second child, we removed the intermediate metatarsals, confirming the appropriate articulation of the medial metatarsal that was saved with the cuneiforms. We reduced the gap between the metatarsals approximating them with a tight surgical stitch, similar to the approximation of ribs, in cases of removal of ribs in scoliosis surgery (Figure 17).

**Figure 18:**
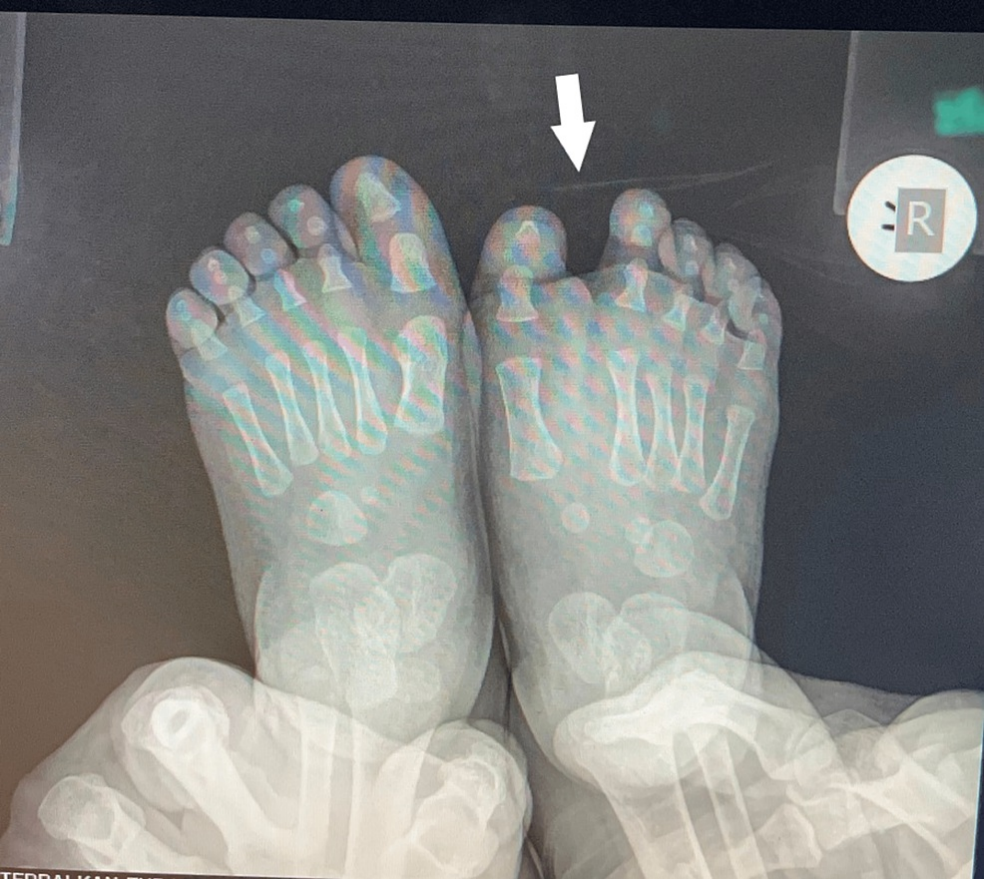
X-ray of the fifth patient's foot six months after the procedure.

**Figure 19:**
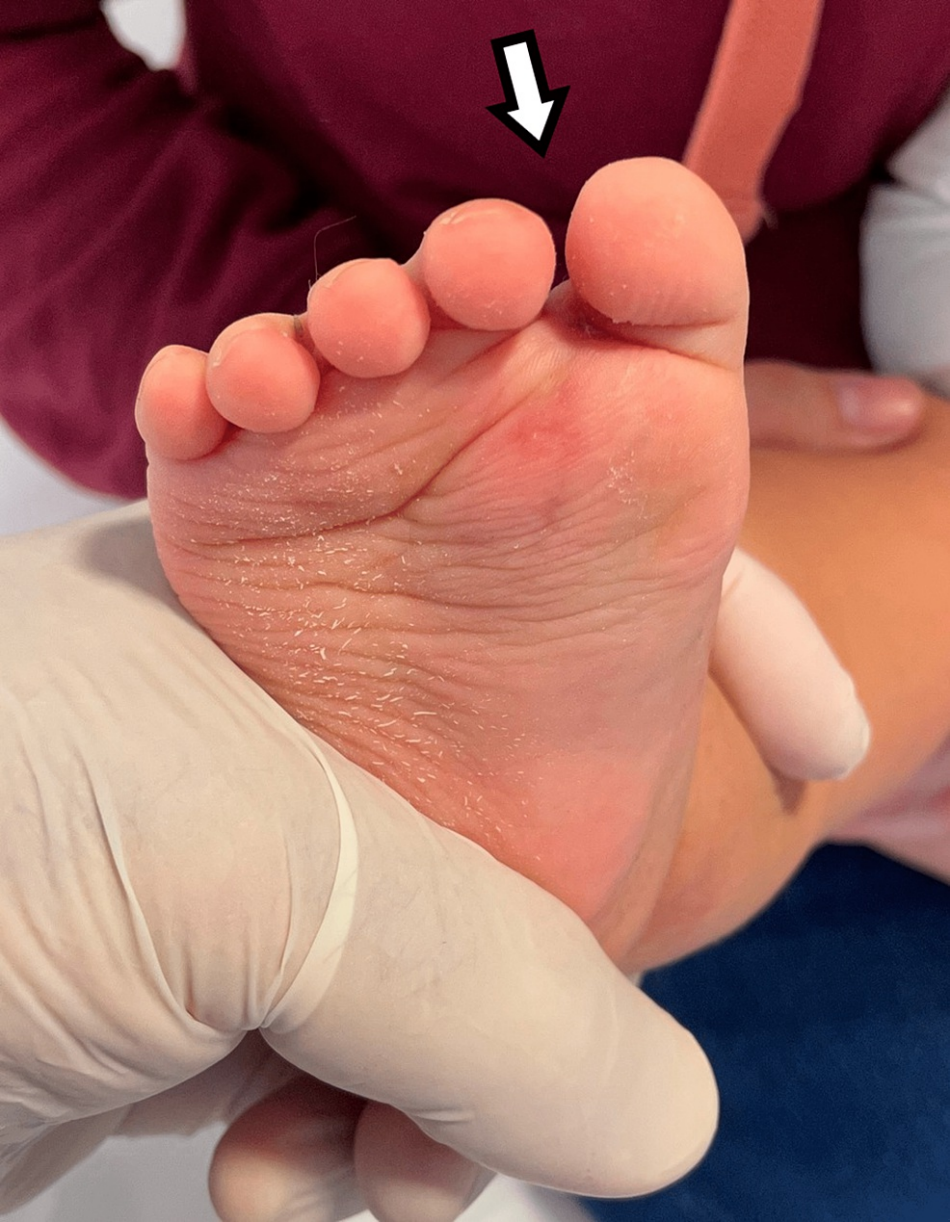
Plantar view of the fifth patient's foot six months after the operation.

A similar procedure was performed for the fifth child, but we used a K wire to stabilize the metatarsal to the cuneiform, which was articulated with the removed metatarsal, and we had to position the remaining medial metatarsal to the cuneiform (Figures 18, 19).

In all procedures, extensors and flexor tendons of the supernumerary rays were found normally developed, with bifurcation in the area of the duplication, and were severed by stitching together their proximal remnants (flexors to extensors).

The child with the bilateral seven toes was easily treated with the removal of the supernumerary fifth toe, with the appropriate trimming of the lateral side of the distal metatarsal. For the great toe, on the right side, we corrected the varus position of the distal duplicated phalange, using a K wire and appropriate skin graft for the corrected medial skin crease, while on the left side, we removed the fused racket type phalange, with correction of the nail bed. On the left side, the skin presented signs of superficial infection for a period of two months that was treated with local cleaning and subsequently subsided.

**Figure 20:**
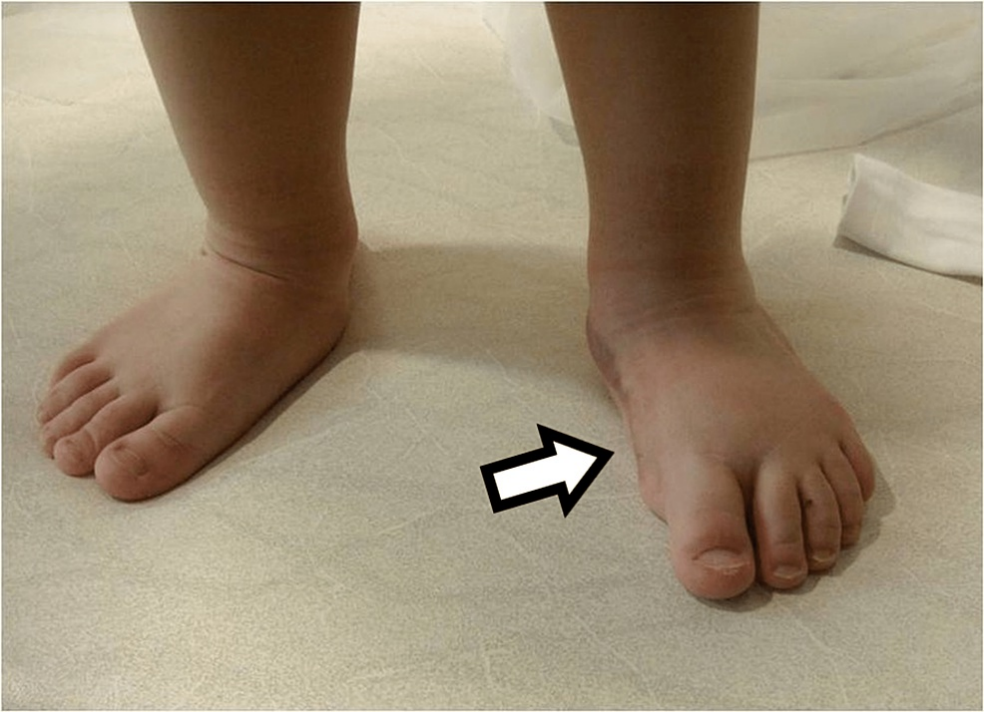
The clinical picture of the foot of the first patient with preaxial mirror foot, nine years after the operation.

**Figure 21:**
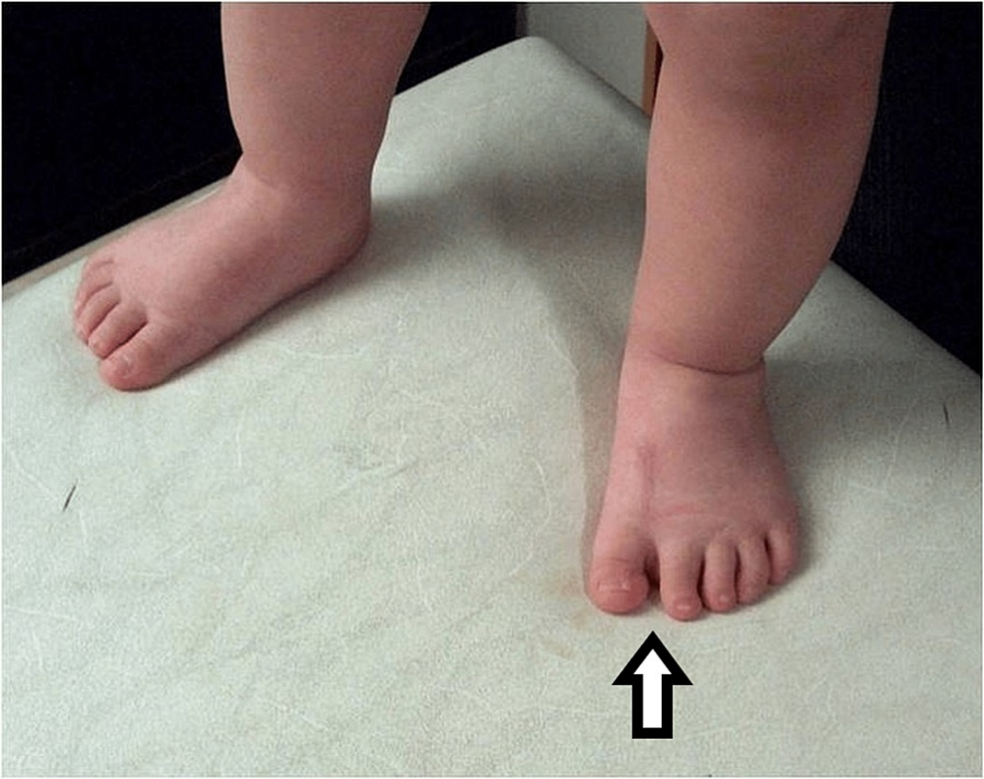
Dorsal view of the second patient with central mirror foot.

**Figure 22:**
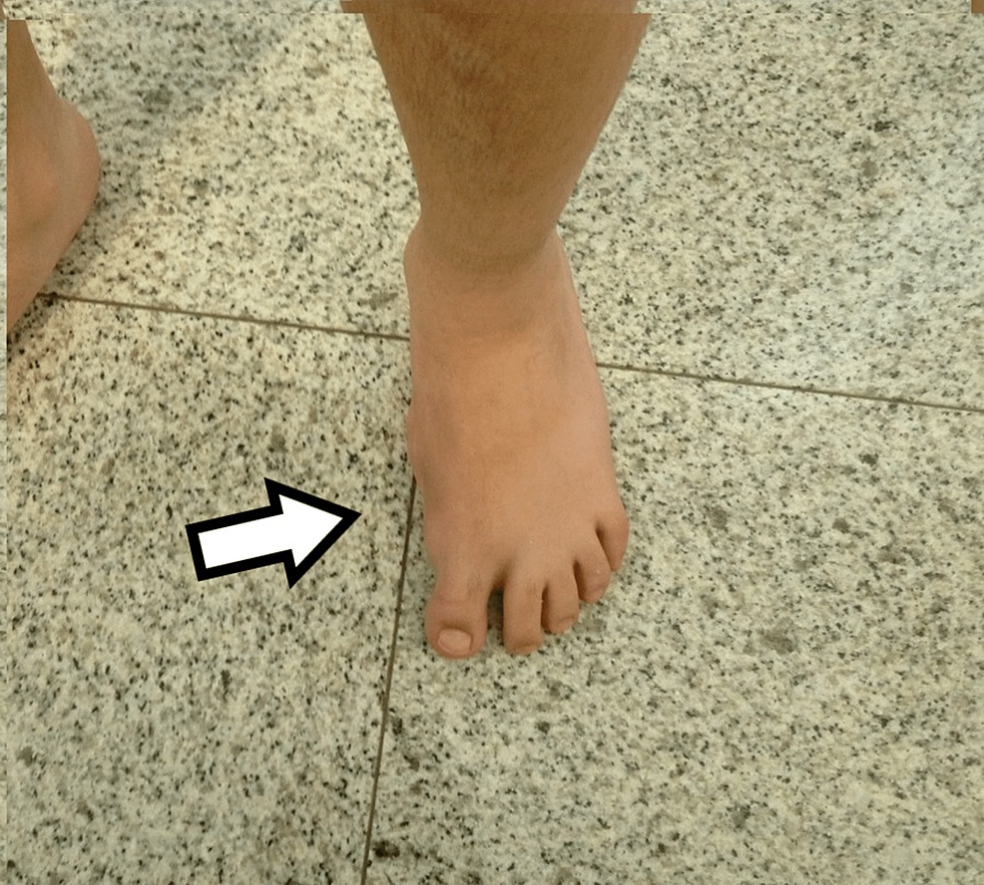
Picture of the foot of the third patient with preaxial mirror foot, eight years after the procedure.

**Figure 23:**
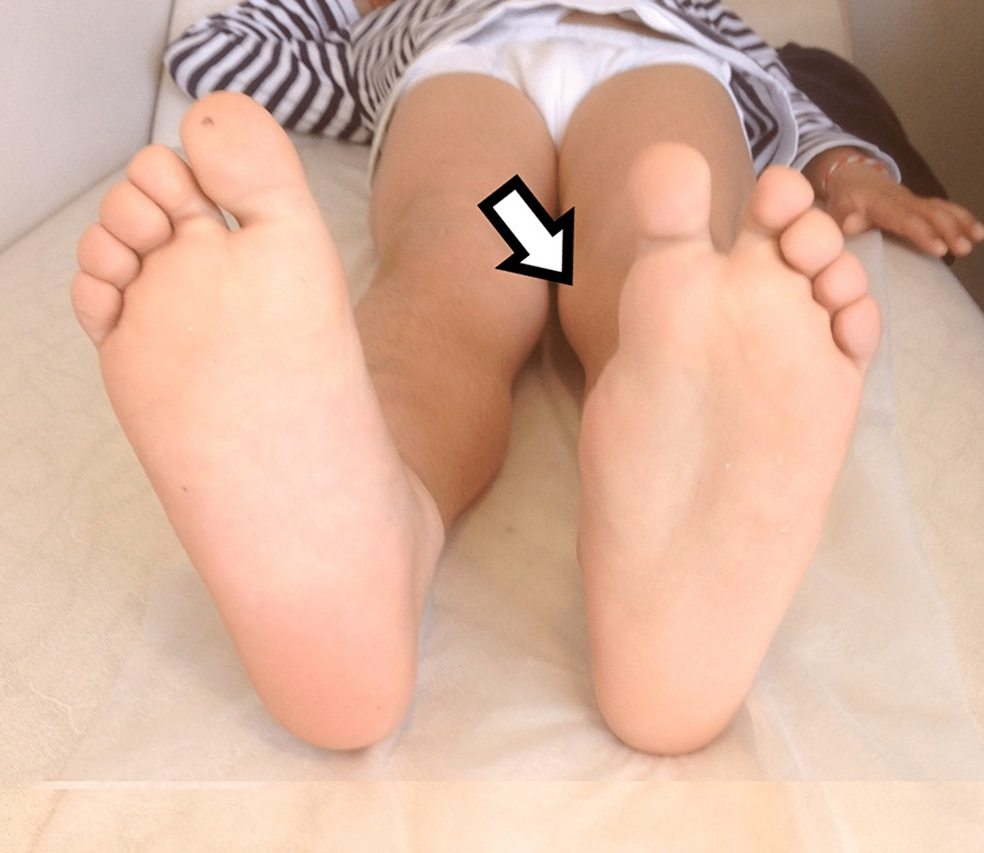
Plantar view of the foot of the third patient with preaxial mirror foot, eight years after the procedure.

We followed up with our patients for a period of 1-9 years. None of our patients have been re-operated. All our patients wear normal shoes and are participating in sports activities (Figures 20-23).

**Figure 24:**
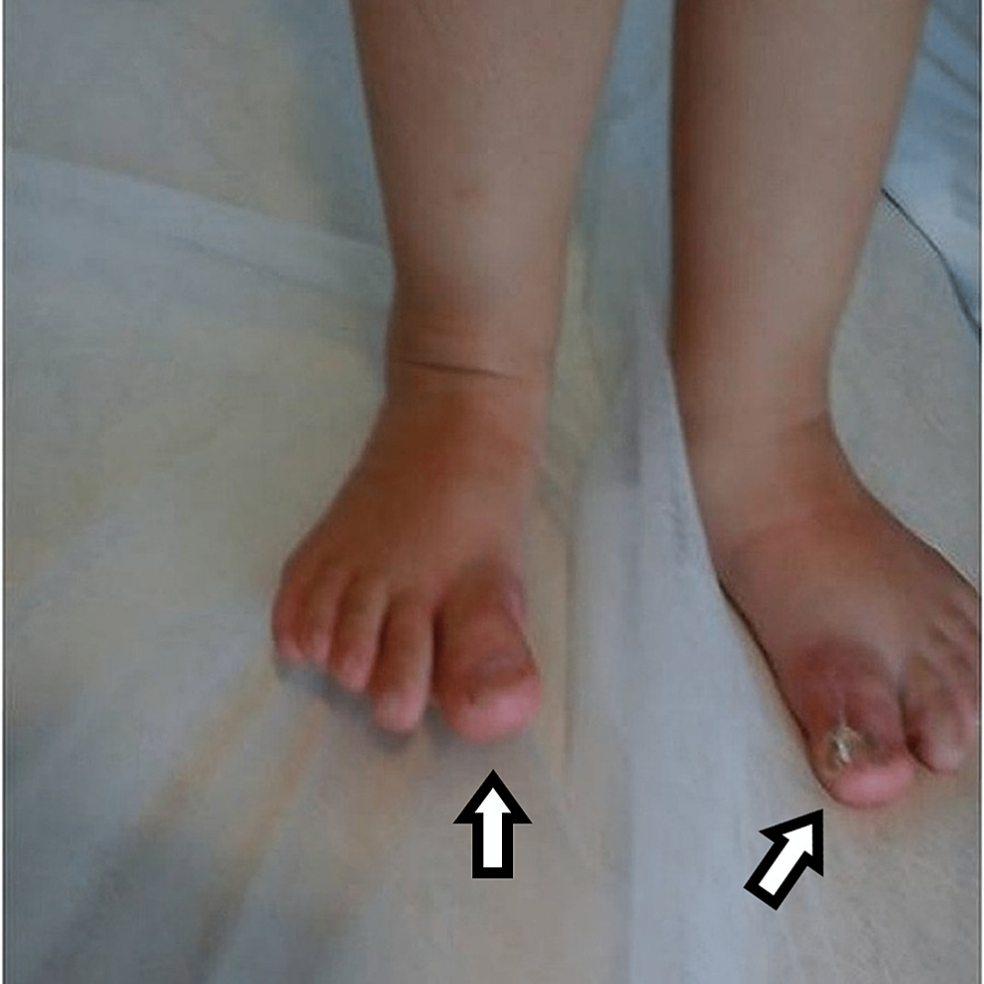
Picture of the fourth child with the bilateral involvement, two years after the procedure. There is mild varus deformity of the great toe on the right foot.

The child with the bracket phalanx still has a moderate varus deformity of the toe that is advised to be treated at an appropriate time with correction of the varus deviation (Figure 24).

## Discussion

Seven-toe foot is an extremely rare dysplasia and with few reports published to date. In this study, we have reported the cases of five children with seven toes and analyzed the various presentations of dysplasia.

Preaxial mirror polydactyly of the foot has been described in association with other dysplasia as fibular dimelia or tibial amelia. It is interesting that is described both with duplication and with the absence of part of the affected limb.

Limb dysplasia may be associated with other dysplasia. Remarkably, none of our patients had alterations of internal viscera. Foot polydactyly has been described in association with vertebral anomalies, as observed in one of the patients [10,11].

Despite that predisposition to polydactyly is considered to have a strong genetic component, none of the patients had a family history of polydactyly. Polydactyly is described as affecting both hands and feet. In the cases observed here, only one patient was unilaterally affected in hand, with a simple floating supernumerary finger on the ulnar side.

Polydactyly is classified as preaxial, central, or postaxial. The vast majority of cases are postaxial (80%). Remarkably, polydactyly with seven toes is either central or preaxial, as was the case for the patients of this study. It is noteworthy that one of the patients showed involvement of the medial great toe and the lateral postaxial toe [1,12].

Dysplasia with polydactyly of seven toes is probably a different type of expression compared with the common six-toe type of dysplasia.

The most common methods of classification of toe polydactyly are the Venn-Watson and the Watanabe classification. Rotterdam foot classification of polydactyly in eight types is based on the level of duplication, from the distal phalanx to the cuneiforms [1,13-16].

Venn-Watson classification describes six types of pre- or post-axial polydactyly according to the duplicated phalanges and the shape and configuration of the metatarsal. Watanabe describes pre-, central, and post-axial polydactyly, further divided into tarsal, metatarsal, and phalangeal groups, according to the level of duplication. This classification is the most applicable in the seven-toe polydactyly described here.

There is no exact definition of the mirror foot. It is reported as a foot with preaxial supernumerary rays with characteristics of post-axial toes, possibly referring to toes with three phalanges.

Fukazawa et al. [2] have proposed a classification of the mirror foot similar to the mirror hand. Comparing the similarities by changing the ulna into a fibula and the radius into the tibia, he proposed five types of deformity. Types 1-3 had either fibula or tibia dysplasia, type 4 is a syndromal foot, and type 5 describes a normal leg with duplication of the foot that includes the hallux.

Considering the Fukazawa classification, patients 1-3 and five can be categorized as type five, except for patient four, whose duplication of the hallux and the fifth toe suggest a different type of seven-foot polydactyly.

Surgical correction aims to restore a normal plantigrade foot with an acceptable aesthetic form that can be easily accommodated in shoes. Several studies report the correction of mirror foot in association with coexistent limb deformities. Verghese [3] observed a series of mirror feet with associated anomalies of fibula and tibia and LLD. Notably, their cases with duplicated calcaneum and cuneiforms were left untreated. Fukazawa reported on the management of three children with mirror feet performing medial ray excision of the supernumerary digits. Conversely, the patients in our study had normal limbs and a normal plantigrade shape. Surgical treatment was solely driven by aesthetics and the associated shoe wear problems.

We have performed medial ray excision in the child with the central ray mirror foot since the most medial ray was the hallux, and the appearance of the X-ray confirmed the polydactyly of the metatarsals. We used a dorsal and plantar incision, removing the metatarsal but without removal of the cuneiforms. The tibialis anterior was found as a single tendon. We approximated the remaining first to the fourth metatarsal using a non-absorbable stitch, similar to the approximation of the ribs in cases of resection in scoliosis surgery. A similar approach was described where the authors used a metal wire [17]. In another case of central polydactyly with eight rays, the authors present an important gap in the shape of the foot after the removal of three rays [18]. Initial removal of the cuneiforms may be an option to reduce the width of the foot.

In the surgical intervention of the first, third, and fifth patients, a medial incision was employed to remove the medial rays, and the accessory navicular with the tibialis posterior was left in place. Mishra and colleagues [5] described a similar case of tendon duplication. Their surgical interventions removed the medial rays of the mirror foot using a medial longitudinal incision early in life, before the ambulation period. Our approach advocates for interventions after one year of age due to the difficulty of accurately assessing the duplicated bones earlier in life, particularly the still unossified cuneiforms.

Children are closely monitored post-intervention since late foot reconstruction in the event of varus deformity may be required. Vlahovic et al. [19] described the severe varus deformity of the remaining medial ray in a nine-toe mirror foot. At the age of nine, the patient was surgically re-intervened to remove the supernumerary tarsal bones and realign the medial ray. We performed monitoring from 1 to 9 years without the need for re-intervention in any of the patients. Removal of the accessory navicular to reduce the width of the foot was suggested for a patient. Nonetheless, the patient feels comfortable with the current appearance and activity of his foot and is reluctant to additional procedures. Further correction of the varus deformity for the patient with the bracket-type of phalange is scheduled later in our treatment plan. Burger et al. advocated the removal of the lateral hallux in cases of preaxial polydactyly with six toes, preferable to the removal of the medial hallux [16].

Lale et al. [20] described the LLD that may appear later in life in children with mirror feet. Deformity of the talus was also reported, causing pain and stiffness in the ankle joint. Remarkably, neither LLD nor talus deformity was observed in the patients of our study.

## Conclusions

This study reports five non-related patients with a seven-toe foot of various anatomical presentations. The patients did not present associated dysplasia in the tibia or fibula, and none of them had been diagnosed with a specific syndrome. Surgical management with the removal of the appropriate rays enabled us to achieve a satisfactory result. The relevance of these cases is critical: If the deformity is recognized prenatally, clinicians can inform the parents about the surgical treatment and its curative effect.
